# The Development and Piloting of an Early Youth‐Engagement (EYE) Model to Improve Engagement of Young People in First Episode Psychosis Services: A Mixed Methods Study

**DOI:** 10.1111/eip.13623

**Published:** 2024-10-22

**Authors:** Kathryn Greenwood, Ruth Chandler, Kirsty Labuschagne, Emmanuelle Peters, Katie Alford, Richard de Visser, Andy Field, Luke Slater, Philippa Garety

**Affiliations:** ^1^ School of Psychology University of Sussex Brighton UK; ^2^ Research and Development Department Sussex Partnership NHS Foundation Trust Worthing UK; ^3^ Department of Psychology, King's College London Institute of Psychology, Psychiatry and Neuroscience (IOPPN) London UK; ^4^ Biomedical Research Centre, Kings College London Institute of Psychiatry Psychology & Neuroscience London UK

**Keywords:** early intervention in psychosis, mixed‐methods, pilot study, psychosisdisengagement

## Abstract

**Aim:**

Psychosis is associated with significant health and societal costs. Early intervention in psychosis services (EIP) are highly effective in promoting recovery, yet substantial proportions of young people disengage. The current study aimed to develop and evaluate a novel engagement intervention in EIP services.

**Method:**

A qualitative investigation of facilitators and barriers to engagement in 68 first episode psychosis patients, family members and young people, and a Delphi consultation with 27 regional and national youth and psychosis service leads informed the development of the intervention.

A mixed‐methods feasibility‐pilot study then compared engagement outcomes in 298 EIP service users in two cohorts: standard EIP versus standard EIP plus the novel early youth‐engagement (EYE) intervention. A qualitative study explored intervention experiences in 22 randomly selected service users, carers and clinicians. A process evaluation explored delivery.

**Results:**

Disengagement was 24% in the standard EIP cohort compared to 14.5% in the standard EIP plus EYE intervention cohort. A 95% Bayesian credibility interval revealed a 95% probability that the true reduction in disengagement lay somewhere between 0% and 18%. The number needed to treat was 11, 95% CI [5, 242]. Use of the EYE resources was associated with engagement. Qualitiative feedback supported effects on communication, social network engagement, service user goals, mental health and well‐being outcomes.

**Conclusion:**

The EYE intervention was designed from a service user, young person and carer perspective. Both qualitative and quantitative data support impacts on engagement. We now need to evaluate effectiveness, cost‐effectiveness and implementation in a multi‐site randomised controlled trial.

## 1 Introduction

Psychosis is associated with poor quality of life, high disability adjusted life years (Rossler et al. 2005), shorter life expectancy (Parks et al. 2006) and high societal costs (The Schizophrenia Commission 2012). The 2–3 years post‐onset are pivotal to long‐term trajectories (Birchwood, Todd, and Jackson 1998; Birchwood and Fiorillo 2000; Bottlender et al. 2003; Pelosi and Birchwood 2003; Harris et al. 2005). Early intervention in psychosis (EIP) services lead to fewer symptoms, relapses and suicides, better well‐being and functioning (Nordentoft et al. 2002, 2004; Craig et al. 2004; Garety et al. 2006; Dodgson et al. 2008; Harris et al. 2008; Melle et al. 2004, 2006; Pinfold, Smith, and Shiers 2007; Lester et al. 2009; Joseph and Birchwood 2005), and are cost‐effective (McCrone et al. 2010; McCrone and Knapp 2007; Knapp et al. 2014; Tsiachristas et al. 2016). Recent standards in England, require that at least 50% of new first episode psychosis patients are engaged with a NICE‐concordant EIP service within 2 weeks (NHS England 2016). Yet, 25%–30% of young people disengage from EIP services within the subsequent 12 months (Doyle et al. 2014). Disengagement has been linked to younger age, male gender, ethnic minority status, substance use, violence, homelessness, milder symptoms, a ‘sealing over’ coping style, limited family contact, lower social function and lack of knowledge of services (Krstev et al. 2004; Schimmelmann et al. 2006; Cotton et al. 2009). Recommended strategies to promote engagement have included targeting groups at high disengagement risk, increasing service assertiveness, improving alliance, satisfaction and perceived need for help, increasing alignment between service user need and service, enhancing collaboration and shared decision making, and providing strong emotional, practical and social support (Kreyenbuhl, Nossel, and Dixon 2009). Yet there is limited evidence for any method to promote engagement, and no study has evaluated a comprehensive intervention to reduce disengagement from EIP services.

This project aimed to identify priority targets to improve engagement from the perspectives of young people and their families; develop a feasible EYE intervention model in collaboration with clinicians and services; and explore the effect of this intervention on disengagement at 12 months post‐referral from EIP services in a pilot cohort study, and on service user, carer and clinician perspectives on the intervention.

## 2 Method

The novel *EYE* intervention was developed through two phases: (i) a qualitative investigation of young person, service user and family perspectives on facilitators and barriers to engagement; (ii) an iterative (Delphi) consultation with clinicians, managers and national leads to translate themes from phase (i) into a consensus intervention model. The intervention was then tested through 3 phases: (i) pilot testing of the *EYE* intervention on disengagement outcomes at 12 months, comparing two service cohorts, one pre‐*EYE* intervention and one post‐*EYE* intervention; (ii) a mixed‐methods process evaluation of implementation from a clinician perspective; and (iii) a qualitative exploration of service user and carer experiences of the intervention. Service user collaboration was central, led by RC, a service user researcher (RC), and supported by a service user support worker, both of whom had lived experience of psychosis. A Service User Research Forum (SURF), comprising service users with differing ethnicities, engagement histories and genders, provided consultation across all phases. Procedures were approved by London‐Dulwich Research Ethics Committee 11/LO/1697 Ref 7471. Written informed consent was obtained from all participants.

### 2.1 Intervention Development

#### 2.1.1 Phase (1) Qualitative Investigation of Facilitators and Barriers to Engagement

Focus group participants were (i) young people aged 16–25 who had been offered or were currently receiving EIP services in Sussex, Surrey or Kent in the UK, (ii) parents and siblings of EIP service users and (iii) young people aged 16–25 in the same regions who were not currently engaged with mental health services but might have need now or in the future, recruited from schools, colleges, YMCA and homeless hostels. People were excluded if they were in receipt of mental health services (young people) or were part of the pre‐EYE project cohort study (a small sub‐group of current service users whose engagement was assessed before the intervention study commenced. They were not invited to focus groups to maintain independence between study phases).

Forty‐five service users, 34 relatives and 25 young people were invited and 22 service users, 24 relatives and 22 young people took part in focus groups or interviews. See Table 1 for participant demographics.

**TABLE 1:** Participant demographics.

Variable	*n*	Age years mean (SD)	Gender % female, *N*	Ethnicity % white british, *N*
Phase 1 Qualitative study	68			
Service users	22	21.06 (2.0)	54% (12)	72.7% (16)
Carers	24	43.82 (11.7)	66.7% (16)	87.5% (21)
Young people	22	21.29 (1.9)	63.6% (14)	77.3% (17)
Phase 2 Delphi study	26			
National EIP leads	2	61^a^	50% (1)	50% (1)
Managers/directors	4	47.3 (5.5)^a^	75% (3)	75% (3)
Team leaders	5	43 (8.8)^a^	80% (4)	100% (5)
EIP clinicians^b^	12	43.4 (8.5)^c^	42% (5)	92% (11)
Support/youth workers	2	43 (21.2)	0% (0)	50% (1)
Pharmacist	1	31	100% (1)	100% (1)
Phase 3 Intervention study	298			
Standard EIP cohort	188	24.03 (0.39)	116 (62%)	—
EYE cohort	110	22.09 (0.50)	72 (65%)	—
Phase 3 Qualitative investigation	22			Gender of carer's service user‐% female (*n*)
Service users	7	—	14% (1)	—
Carers	7	—	100% (7)	57% (4)
Clinicians	8	—	75% (6)	—

^a^ Data missing for one person.

^b^ EIP clinicians comprised 5 psychiatrists, 2 psychologists and 5 care co‐ordinators (nursing, OT and social work).

^c^ Data missing for 3 people.

Focus groups were facilitated by a research clinician (KG), a research assistant (KL) who also conducted all individual interviews and either the service user RC or a service user from the SURF group who was male and from an ethnic minority. Focus groups and interviews followed a semi‐structured format and explored facilitators, barriers and service recommendations to improve engagement. They were audio‐recorded, transcribed and analysed using Thematic Analysis (Braun and Clarke 2006). A multiple‐coding approach was used to reach consensus on themes for development of the intervention model: Initial rich transcripts in each participant group were coded by all three researchers (KG, RC, KL) and subsequent selected transcripts were also reviewed. Group discussions informed the early coding frame and final consensus on themes and quotes. These were compiled into two booklets outlining barriers and facilitators to engagement, and shared in the Delphi focus groups.

The results revealed 5 core themes: (i) Communication—amount, style and content; (ii) Social involvement—family, friends, peers, media; (iii) Mental Health Service—approach to admissions and medication; (iv) Staff—knowledge, attitudes and behaviour; and (v) Personal experience—mental health, relationships and experiences that could affect engagement. The detailed content of these themes will be reported elsewhere.

Phase (2) Delphi consultation.

An iterative Delphi consultation (e.g., Marshall et al. 2004; Morrison and Barratt 2010) was employed with clinicians, managers and national EIP service leads, to reach consensus on the engagement intervention model derived from the phase (i) themes.

First, themes and quotes from phase (i) were presented in a series of focus groups to generate a list of 44 potential intervention approaches. Next, participants provided comments and rated the importance and feasibility of each approach on a 5‐point likert scale from not at all important/feasible to very important/feasible. Finally, participants reviewed their own and summary ratings (% endorsing each point on the likert scale and written comments) and made final ratings.

Intervention approaches were incorporated into the EYE engagement model if they were rated as ‘Very Important’ by the majority of participants (over 50%) or were rated as ‘Important’ (by over 80%) and feasible (‘Very Feasible’ by over 50% or ‘Feasible’ by over 80% of participants). The SURF group reviewed potential intervention components and those that were rated not important or feasible. They requested that social groups were reintroduced prior to the final Delphi consultation, and social groups were approved in the final consensus.

### 2.2 Final EYE Intervention Model and Training

The final EYE intervention model comprised (i) a staff training package supported by a draft manual and training videos focussed on therapeutic communication and including motivational approaches, open transparent, goal‐focussed communication and collaborative treatment choices in the context of risk and life‐cycles, co‐led by service users and carers; psychoeducational resources to support this communication and address personal barriers including (ii) the psychoeducational series of “myth‐busting” information booklets about mental health and help‐seeking, EIP services, treatment choices and advice for friends and family; (iii) posters for GP surgeries; (iv) the engagement website with information, videos, recovery stories, forums, help‐seeking advice, self‐help information and schools pack; and a social (systemic) engagement approach including (v) a broad family and friends social involvement protocol; (vi) social groups; and (vii) access to peer support workers. SURF members were involved in developing the intervention materials and approaches. They co‐wrote and edited the psychoeducational booklets and website, took part in photo‐shoots for materials and trained as peer workers, delivering peer support sessions and co‐led social groups to EIP service users. The intervention addressed themes from phase (1) and included intervention approaches from phase (2).

Training in the EYE motivational‐engagement intervention was a whole day programme, co‐delivered by SURF members, carers, the research team (KG, RC, KL) and a motivational‐interviewing trained therapist. RC and the SURF group wrote and produced two staff training videos based on the phase 1 interviews, to demonstrate good and poor engagement, and used these in a service user led training session. A carer and RC, who also had caring experiences, co‐delivered training on carer involvement. The manual included (i) an introduction to the study and the intervention, (ii) a description of the components of the EYE intervention, (iii) the training slides and supporting documents and (iv) a complete set of EYE resources. Each team received a training consolidation meeting in the subsequent month, and additional consultation as required thereafter. Over 85% of all EIP staff attended the training.

### 2.3 Intervention Testing

#### 2.3.1 Phase (3.I) Pilot Testing of the EYE Intervention

Participants for the pilot study were two complete cohorts of all young people (see Table 1 for demographic data), referred to specific EIP teams in Sussex and Kent, during two designated periods: one prior to intervention development, (the comparison group), who were referred over 10‐months in Sussex or 7‐months in Kent; and one following training and implementation of the intervention 21 (Sussex) to 27 months (Kent) later, (the intervention group), who were referred over 7 (Sussex) or 4‐months (Kent) and received standard EIP plus the new EYE motivational‐engagement intervention. All new EIP patients referred during each period were included provided they met entry criteria for the service (definite or probable psychosis based on all available information). Participants were excluded if (i) they entered the service after receiving treatment from another EIP service (defined as at least 3 service contacts over at least 3 months duration), or (ii) they moved out of area during the follow‐up period and engaged with a new local service. An a priori power calculation revealed that for a minimum clinically significant 50% reduction in disengagement from 30% in the comparison cohort to 15% in the EYE intervention cohort, with alpha at 5% and power at 80%, 242 participants would be needed.

The primary outcome was the proportion of service users who disengaged at 12‐months, defined as refusal to engage with EIP service, or lack of response to all EIP contact over a consecutive 3‐month period. A secondary quality of engagement outcome was rated by care co‐ordinators using the Health of the National Outcome (Engagement) scale (HONOS, Wing et al. 1998), expanded to capture problematic disengagement (−4); optimal (0 score); and problematic over‐engagement (+4).

The intervention study compared disengagement at 12‐months for 298 participants, either in the comparison cohort (*n* = 188) or the EYE cohort (*n* = 110). A generalised linear mixed model (GLMM) was fitted to the primary categorical disengagement outcome using the glmer() function in the lme4 package in R (Bates et al. 2015). For the secondary continuous engagement outcome, the lme() function from the nlme package was used (Pinheiro et al. 2013). The core multilevel model with observations (level 1) nested within team (level 2), was built up as follows: intercept only model; intercepts allowed to vary across teams; main effect of cohort added (EIP service vs. EIP plus EYE intervention); random slopes for cohort effect across teams; effect of covariates (age/gender). Where possible bootstrap confidence intervals were calculated. Credibility intervals for the key effects were estimated using Bayesian methods adopting a uniform prior distribution.

#### 2.3.2 Process Evaluation of Implementation of the Intervention

A small‐scale process evaluation investigated implementation of the intervention, in terms of clinicians' activities and attitudes towards the intervention, resources and training. All clinicians with at least one service user in the EYE intervention cohort were invited to complete a brief questionnaire concerning the resources and approaches used, and the impact on their work. A generalised linear mixed model (GLMM) was fitted using the glmer() function in the lme4 package (Bates et al., 2015), with engagement scores nested within sites, random intercepts, fixed effects of the specific outcome (training, social involvement approach, communication approach) and random slopes across sites, retaining the most parsimonious model.

#### 2.3.3 Qualitative Evaluation of the Experience of Implementing and Receiving the Intervention

The experience of implementing and receiving the EYE intervention was explored qualitatively in 8 clinicians, 7 service users and 7 carers, randomly selected, stratified by team, from the intervention cohort to provide feedback in individual interviews regarding the intervention delivery and its impact. Interviews were conducted individually by a Research Assistant (KL), independent of the EIP service, to facilitate openness and honesty, and were audio‐recorded, transcribed, analysed thematically using a multiple‐coding approach (Braun and Clarke 2006). Selected transcript in each participant group were coded by three researchers, (KG, KL, RdV) and the lived‐experience researcher (RC), initially and mid analysis, and discussed in group meetings to reach consensus on the coding frame and themes. The data were analysed separately in each participant group and combined to form final themes.

## 3 Results

### 3.1 Pilot Testing of the EYE Intervention

There were no significant differences between cohorts in terms of gender (χ2(1) = 0.42 *p* = 0.52), or substance use (at baseline (χ2(2) = 2.98 *p* = 0.23) or follow‐up (χ2(3) = 2.46 *p* = 0.48)) where documented, *n* = 212). The EYE cohort was slightly younger than the standard EIP cohort (Ty = 3.04 [0.73, 3.45] *p* = 0.004). A generalised linear model with engagement status nested within team, revealed that the proportion engaged was significantly higher in the EYE intervention cohort compared to the standard EIP cohort. Engagement was not predicted by team, age or gender (Table 2).

**TABLE 2:** Results of the generalised linear model to investigate the impact of cohort (EYE model), team, age and gender on disengagement outcome at 12 months.

Model	Df	AIC	BIC	LogLik	Deviance	Chisq	Df	Pr (> Chisq)
Random intercept	2	305.98	313.38	−150.99	301.98			
Cohort	3	303.69	314.78	−148.84	297.69	4.292	1	0.038
Team	5	307.59	326.07	−148.79	297.59	0.105	2	0.95
Age and gender	7	309.85	335.73	−147.93	295.85	1.732	2	0.42

Abbreviations: Df = Degrees of freedom; AIC = Aikake Information Criterion; BIC = Bayesian Information Criterion.; LogLik = Log Likelihood; Chissq = Chi‐squared; Pr = probability.

As the effect of the EYE intervention did not differ by team, and no other predictors were significant, a more parsimonious model with only cohort as a predictor of engagement was maintained. Disengagement decreased from 24% in the standard EIP cohort to 14.5% in the EYE intervention cohort (see Table 3). A 95% Bayesian credibility interval suggested there was a 95% probability that the true reduction in disengagement lay somewhere between 0% and 18%. This means that at the upper limit, the EYE intervention could have a fairly‐large effect, with 18% of service users prevented from drop out, and at worst will do no harm.

**TABLE 3:** Number of service users engaged and disengaged at 12 months, in the standard EIP cohort and the EYE intervention cohort.

Cohort	Engaged	Disengaged
Standard EIP	143	45
EIP plus EYE intervention	94	16

The number needed to treat was 11, 95% CI [5, 242], suggesting that for every 11 people who received the EYE intervention, one person would be prevented from dropping out.

The quality of engagement, as rated by the care co‐ordinator, did not differ significantly by site, or by intervention cohort (EYE vs. standard EIP), and there was no interaction. Adding gender and age to the model did not improve its fit. The final quality of engagement model with random intercept and slope, showed no significant main effect of cohort (*t* = 1.17 *p* = 0.24, bootstrap 95% CI [−0.160, 0.665]).

### 3.2 Process Evaluation of Implementation of the Intervention

Care co‐ordinators (*n* = 80) completed a brief questionnaire on their use of the intervention for a subset of 93 (of 110) service users. Eighty responded about their use of social involvement approaches, 75 about their use of resources and 52 about the impact of training. Ratings of training and social involvement did not significantly influence disengagement. However, the more EYE communication approaches used (booklets and website) to engage participants, the more likely participants were to be engaged 12 months later (Estimated effect −0.66; *p* = 0.014). The Bootstrap 95% CI for the effect of communication approaches suggests that, if we assume this sample is one of the 95% for which the CI contains the population value, then using one additional communication device would give rise to a decrease in log non‐engagement of between −1.89 and −0.23. However, missing care co‐ordinator ratings of 27%–51% for some components mean that results should be treated with caution.

### 3.3 Qualitative Evaluation of the Feasibility and Experience of Implementation

The qualitative study revealed that the EYE model was feasible, well‐implemented and positively received by service users, carers and clinicians. Ten themes were identified (see Table 4) relating to (i) Communication, (ii) choice and collaboration, (iii) staff approaches (iv) a shared focus on practical goals (v) social involvement, (vi) feeling more hopeful, (vii) feeling more trust and honesty (service users), mirrored by clinician transparency (viii) feeling more validated and reassured (carers) and confident in the quality of information received, (ix) feeling more pride and professionalism in the service (clinicians) and (x) feeling less isolated and stigmatised (service users). Service users and staff felt that the resources supported the EIP approach and worked best when used in joint sessions to guide collaboration and treatment plans.

**TABLE 4:** Example quotes to support the themes arising from the qualitative thematic analysis.

Communication Carer: Well I think it looks pretty useful [youth mental health booklet]…it's actually got some of the answers to some of the questions I have! Carer: I'm pretty sure he has googled about psychosis so you really wanna make sure the information you are getting is researched and not just google rubbish. Carer: If it was available to people, initially, especially when they first end up in hospital. That would've made so much difference. [Service user] always said she felt different…even when she was young. Something like [the booklets] could change the whole outlook couldn't it. Care Co‐ordinator (CC): The first booklet, mental health and getting help, I normally give them one of those to begin with and then if it's obvious that we're looking at first episode psychosis then they get the next book. So that's been really helpful. It's nice to actually give somebody something to go away with to read rather than just assessing somebody for a possible first episode of psychosis and then sort of just going on their merry way. It's quite daunting but if they've got something they can actually read and, perhaps find a part, questions they wanna ask, it helps for the next stage. CC: The friends and family booklet, is worth its weight in gold, cos at the point people are referred to our service, Mum, Dad, whoever, are very very stressed and often pulling their hair out and often, need a lot of support. So just to have that, and this is from feedback from mums and dads, there's something actually written for me. I'm such a big part of this person's life and a big part of their experiences as well, that it's nice that they feel a bit acknowledged, that time and effort has gone into really trying to understand where they're coming from. CC: I have found the leaflets and referring to the website has been helpful because it kind of backs up the message that we try and get across to people, their carers and friends Service User (SU): I wish I had received [the booklets] at the beginning, everything is very clear and knowing what different people do and what to expect for EIS … really good to have that written down.
2 Choice and collaboration Carer: She decided she was coming off [the medication], which was all a bit scary for everyone, and they were great actually then. They still listened to her to the fact that she wasn't gonna take it. And although we all thought she was just gonna go straight backwards, she didn't really. CC: It's a really good way of opening up a discussion about all these things that we know clients and families worry about … it's really good to kind of have them all in one place. It very much feels collaborative with the client, their family and friends when we're sat around together looking at these things. CC: We talk about their team. Their team isn't just me and the doctor and the psychologist, it's also their best friend and their mum. We share things like relapse prevention plans with that person's micro team. CC: Families are being routinely more involved in decisions CC: At the point we take someone on, giving something like an interventions booklet helps guide their thinking as well as ours… SU: It doesn't feel like they're nudging or anything, or pushing me towards something. There's a lot of encouragement to do more things. But it's more like I'm still, the one that's kind of in charge.
3 Staff approaches Carer: [EIP] try and get her to engage with them and let her know that they are there, so that you hope that if there's a flash of light or something, that she might, and occasionally she does, ring them
CC: My initial contacts with clients are now much different since the introduction of the EYE materials. They very much form the basis of initial discussions with clients, their friends and carers and relatives. It's quite a positive development in terms of that initial engagement. CC: I think something has been shifting in the [other staff] I've been working with. I think it's something around the ethos about EI being talked about a lot more regularly and dispelling myths—I think funnily enough the identity of EI has been a bit more enforced. SU: They just seem more caring, it's like they understand, cause before I didn't think anyone would understand what it was and then when [care co‐ordinator] was explaining it to me, like the other people that have this or that and they do certain things—it just made me feel much better.
4 Shared focus on goals Carer: Sometimes, her ideas are quite out there, but they don't discourage… and they've set the small goals you know like, taking her to a coffee shop. She's gone from not going out at all, to the poetry you know. CC—I ask a lot of people about their goals, aspirations and we work towards that. CC—I have one young man who is incredibly difficult to engage, monosyllabic. There is an element of me needing to be a bit more intervention focused and that's when I thought, that's quite a good idea, [to give treatment choices booklet] cause that's all the stuff we could be doing. SU: They do try and find out what I want to do, and give me booklets about courses and stuff. I know that there's an occupational therapist there, if I need to speak to her. I know that all the support's there.When I'm ready to engage then I will.
5 Social inclusion Carer: We all went and we all sat there and gave our input of what we thought might be the problem … they have been tremendous. CC: I'm actually starting up a young people's social group. It's gonna be a social inclusion group rather than a mental health group, focusing on leisure stuff and social activities and part of that is inviting friends. Erm, so if they've got a boyfriend or a brother or a sister or a friend they can bring them. I'm hoping from there, I'll be able to find more about their friends and, you know how much support network people have got. CC: It does get them looking at the website … I think that's been really helpful as a cue to say you're not the only one out there, it's actually quite common and, you know, an awful lot of people have these experiences. CC: One of my clients has been involved recently with the peer worker and in developing a poster to start a social group SU: He's really pushed me to see my friends, yeah, he always tries hard. SU: I suppose it's nice to actually hear that someone else has gone through these crazy thoughts
6 Hope Carer: I did like the quotes from other carers and young people.It was nice to know that some people actually do get better! CC: The hope talk I think that one of your colleagues gave was very interesting. A lot of the time now, actually in conversations with our clients, we talk about hope and we'll literally use the word hope. Specifically that actual word comes into conversation a lot. SU: I might get another chance. I'm hoping they have some sort of courses or something. I just need like a little gap, a little opening sort of thing.
7 Trust and transparency CC: It's nice to have quotes [in the booklets] by different people who've had different treatments and they've said something about it. I think that's nice for information and makes it maybe a bit less clinical and a bit kind of sort of connects with people more. CC:—It's very obvious that it's been developed with service users ‘cos the questions specifically about medication and treatment are exactly what clients say to us all the time, pre the birth of the EYE project. SU: Yeah, he's good to get on with. I mean, I speak to him on a level…[I'm] honest with him as well. I'm not worried. [He] has explained to me like if I do start feeling unwell, he said I won't go straight back to hospital …it's the last thing they do whereas before it was the first thing they did
8 Reassurance CC: Carers are the unsung heroes aren't they. So anything we can do to support that engagement with families … anything that will aid clarity of understanding and help people feel that they're better able to support their loved one. Carer: If I'm feeling quite miserable about (SU), just by reading them [EYE booklets], they're only little pamphlets but I might just flick through them and think you know this is to be expected, this is confirming it … Reassurance… I do like that, and with the do's and don'ts … I'll just have a little look at them and I can think yeah I am doing exactly what they say to do
9 Pride and professionalism CC: The service user involvement is always powerful isn't it? And that was great to have that, [in the training]… it's something you see less CC: The booklets are much more professional than giving out silly bits of paper, print outs you know which were just naff—it's much more up‐to‐date CC: It kind of makes us look more professional…it's frustrating when people who work in private and corporate [institutions]… have greater access to more engaging and more dynamic literature. So, I think it puts us more on a level par with what younger people are used to receiving.
10 Reduced isolation and stigma Carer: [with social groups] It's building the confidence to go and try these things in a safe situation and then you can go on to do that on the outside CC: In the psychosis booklet, it says about, they wanna put everyone in hospital, and then there's an answer. Actually no we don't want to do that. I think that's quite, empowering, quite positive that…we don't want there to be a stigma in mental health. CC: With peer workers, we would be using him quite a lot cause it's very anti‐stigmatising … a lot of the people I've got have come in after a long admission and don't see a future…[peer workers are] more effective than half of us clinicians. CC: 3 o'clock in the morning, not sleeping with stuff going on in their head then they actually have something to turn to. It's much better than ringing help lines … looking at other people's experiences, listening to other people's experiences. I think you know it does help with that isolation… CC: I explain, what the service is like, you know, working with young people who experience unusual experiences other people don't, such as, hearing voices, believing things others might not believe. I do it quite loosely, Again it's not feeding into the stigma but it's trying to make people feel at ease SU: You don't want people to know you got a problem cause of what you said, like stigma and that, so from what I read I think it would be good to see these [booklets] if you weren't feeling well. The information in there is good and I like hearing what other people have got to say. SU: I mean…there was times when I got worse and I needed information to kind of help me to feel like I wasn't alone. So, from here I did… I typed in, I went on the internet [EYE website], and then, I read a few stories or something. Yeah, It stopped me feeling like I was alone.

Despite these positive experiences, some negative experiences still occurred. EYE resources were not always used or received; ambivalence remained for some service users and clinicians regarding family and social involvement; staff felt that holding hope and maintaining engagement is sometimes hard, and needs on‐going training; and also that service users' initial experiences with other services, and especially hospital admissions, negatively affected subsequent engagement. Social groups and peer worker support were less well‐implemented, and there was consensus across groups, that a less formal approach, with peer support workers in social groups would work best, enabling service users to help others, gain confidence and improve their own well‐being.

## 4 Discussion

Our results showed that disengagement was nearly 10% lower in the EYE intervention compared to the standard EIP service, and the number needed to treat analysis suggested that for every 11 people offered the EYE intervention, one person would be prevented from dropping out. The process evaluation suggested that lower disengagement was associated with greater use of EYE communication resources. There was no difference in the quality of engagement. The qualitative evaluation indicated impacts on communication style, engagement with social networks, focus on service user goals and on mental health and well‐being outcomes including hope, empowerment and trust, isolation and stigma, reassurance for families and pride and professionalism for clinicians who really valued the EYE resources. Thus, not only did the EYE model appear to reduce disengagement, but it may also enhance service user and family mental well‐being, and staff satisfaction.

The intervention was evaluated as a team treatment package, with limited opportunity to explore the effect of specific components, but various components reflect developments in the clinical literature. Treatment choice and shared decision making are becoming critical for psychosis (Morrison et al. 2012; Tyrer 2012). The value of involving friends of young people with psychosis, in their treatment, has been emphasised (Harrop et al. 2015). Qualitative investigations of carer involvement have demonstrated the values but also challenges in supporting carers themselves (Lavis et al. 2015; Hickman et al. 2016). There are models for peer workers in EIP services (Robinson et al. 2010), and work by our group has demonstrated the value of hopefulness on therapeutic relationships and social inclusion outcomes (Berry and Greenwood 2015).

However, not all engagement concerns and approaches were represented in the EYE model. Some concerns such as pressure to accept treatments, were covered by multiple intervention approaches (e.g., communication style, service approach to medication and the treatment choices booklet), whilst others, although important, were not deemed feasible in real‐world settings. These included components such as ‘Providing flexibility regarding who you work with, especially including gender’, which was viewed as infeasible in small services, and ‘Dedicated youth in‐patient beds’, which was beyond the scope of an EIP service adaptation.

It was initially surprising that care co‐ordinator‐rated quality of engagement did not differ between arms. This might reflect a lack of sensitivity of the single HoNOS item, a lack of power, or a lack of impact on clinician‐rated engagement. Literature attests to the distinction between service user and clinician‐rated therapeutic alliance (Neale and Rosenheck 1995; Bale et al. 2006). Indeed, clinician‐rated engagement may overlap with perceived compliance with medications, whereas service user‐rated alliance, may overlap with empowerment, treatment choice and engagement with alternative treatments. The EYE intervention was specifically designed from the service user perspective, and thus it makes sense that the greatest effect relates to service users' engagement perspective; namely, their decision to remain with the service.

At this pilot stage, no consideration was given to cost‐effectiveness which is critical to the implementation of any new service intervention. Key staff training and booklet printing costs are comparatively low, but more work is required to demonstrate this empirically.

## 5 Limitations

The study design was a pre‐post intervention cohort study. This was necessary at this pilot stage where the primary outcome was service disengagement, and the intervention was delivered by the whole team. However, this is not a robust test of the intervention. Other service changes, during the course of the study, and independent of the EYE model, might also impact disengagement. Specifically, small caseload size, a key principle of the standard EIP model (Department of Health 2001), nearly doubled in some teams during the post‐EYE‐model cohort. However, the anticipated effect of increased caseloads would be higher disengagement in the post‐EYE cohort, so this does not weaken, and may even strengthen the conclusions drawn from this early pilot work. Moreover, there were no differences in implementation or engagement outcomes by team, suggesting that the intervention effect was not influenced by team, and could be delivered in a range of service settings. The study incorporated people from minority ethnic populations, but some additional adaptations to resources may be required to better engage more ethnically diverse urban populations. We have addressed these adaptations in a separate study (Rathod et al. 2023). Implementation was not universal for all components and fidelity varied, as evidenced by clinician ratings and qualitative feedback. Finally, some engagement barriers, such as lack of access to EIP services out of hours, negative in‐patient experiences and lack of match between clinician and service user demographics, which were not addressed in the study, may still have affected disengagement.

## 6 Conclusion

Disengagement of young people from EIP services remains an important issue, with rates up to 30% after 12–33 months (Doyle et al. 2014). EIP services have made progress in early detection and rapid provision of NICE‐guidelines concordant services. National policy, investment and service structures have focused on ensuring that young people are proactively engaged in assessment, and offered a full EIP care package to prevent them ‘falling through the gaps’, experiencing poor outcomes and requiring greater subsequent healthcare use (p27; NHS England 2016), but there is limited evidence for methods to promote engagement in the subsequent 3 years. Our work has begun to provide this evidence. We now understand some of the reasons why people disengage. The results of our pilot study are promising in terms of reducing disengagement, with potential to improve service user well‐being, mental health, carer support and staff pride and professionalism. We have developed tailored psychoeducational resources to support EIP services to implement the intervention nationally. We are now evaluating the effectiveness, cost‐effectiveness and implementation of the EYE intervention in a large multi‐site cluster randomised controlled trial (EYE‐2: ISRCTN51629746; Greenwood et al. 2021).

## Conflicts of Interest

Prof Kathryn Greenwood has received an honorarium for an academic presentation from Boehringer Ingelheim Ltd. Professor Andy Field has received honoraria/payments from statistical book publications. Professors Greenwood, Peters, deVisser, Field and Garety and Ruth Chandler have received NIHR and Research Council Funding. No other declarations of interest exist.

## Data Availability

The data that support the findings of this study are available on reasonable request from the corresponding author, (KG). Analytic code is available on reasonable request from the statistical lead (AF) for the purposes of reproducing results. Training materials and booklets are available on reasonable request from the corresponding author (KG) for the purposes of reproducing results.
